# Outpatient Therapeutic Feeding Program Outcomes and Determinants in Treatment of Severe Acute Malnutrition in Tigray, Northern Ethiopia: A Retrospective Cohort Study

**DOI:** 10.1371/journal.pone.0065840

**Published:** 2013-06-06

**Authors:** Henock Gebremedhin Yebyo, Carl Kendall, Daniel Nigusse, Wuleta Lemma

**Affiliations:** 1 Department of Public Health, College of Health Sciences, Mekelle University, Mekelle, Ethiopia; 2 Department of Global Community Health and Behavioral Sciences, School of Public Health and Tropical Medicine, Tulane University, New Orleans, Louisiana, United States of America; Aga Khan University, Pakistan

## Abstract

**Background:**

Outpatient Therapeutic feeding Program (OTP) brings the services for management of Severe Acute Malnutrition (SAM) closer to the community by making services available at decentralized treatment points within the primary health care settings, through the use of ready-to-use therapeutic foods, community outreach and mobilization. Little is known about the program outcomes. This study revealed the levels of program outcome indictors and determinant factors to recovery rate.

**Methods:**

A retrospective cohort study was conducted on 628 children who had been managed for SAM under OTP from April/2008 to January/2012. The children were selected using systematic random sampling from 12 health posts and 4 health centers. The study relied on information of demographic characteristics, anthropometries, Plumpy'Nut, medical problems and routine medications intakes. The results were estimated using Kaplan-Meier survival curves, log-rank test and Cox-regression.

**Results:**

The recovery, defaulter, mortality and weight gain rates were 61.78%, 13.85%, 3.02% and 5.23 gm/kg/day, respectively. Routine medications were administered partially and children with medical problems were managed inappropriately under the program. As a child consumed one more sachet of Plumpy'Nut, the recovery rate from SAM increased by 4% (HR = 1.04, 95%-CI = 1.03, 1.05, P<0.001). The adjusted hazard ratios to recovery of children with diarrhea, appetite loss with Plumpy'Nut and failure to gain weight were 2.20 (HR = 2.20, 95%-CI = 1.31, 3.41, P = 0.001), 4.49 (HR = 1.74, 95%-CI = 1.07, 2.83, P = 0.046) and 3.88 (HR = 1.95, 95%-CI = 1.17, 3.23, P<0.001), respectively. Children who took amoxicillin and de-worming had 95% (HR = 1.95, 95%-CI = 1.17, 3.23) and 74% (HR = 1.74, 95%-CI = 1.07, 2.83) more probability to recover from SAM as compared to those who didn't take them.

**Conclusions:**

The OTP was partially successful. Management of children with comorbidities under the program and partial administration of routine drugs were major threats for the program effectiveness. The stakeholders should focus on creating the capacity of the OTP providers on proper management of SAM to achieve fully effective program.

## Introduction

Severe Acute Malnutrition (SAM) is defined as weight-for-height ratio of less than −3 standard deviations below the median reference population or weight-for-height ratio of below 70% or presence of nutritional oedema [1].

Directly or indirectly, malnutrition contributes to 53% of deaths of children under-five in developing countries. According to the United Nations International Emergency Fund (UNICEF) estimates, around 26 million under five children suffer from SAM in developing counties [2]. Ethiopia is one of the countries with highest under-five child mortality rate, with malnutrition underlying to 57% of all children deaths [3]. The latest Ethiopia Demography and Health Survey stated that stunting, wasting and underweight among under-five children in Tigray region are 51.4%, 10.3% and 35% respectively [4].

Until recently, the management of SAM has been limited to hospital cares with limited coverage [5]. Outpatient Therapeutic feeding Program (OTP) brings the service of management of SAM closer to the community by making services available at decentralized health facilities (primary health care units) in different resource limited countries such as Niger, North and South Sudan, Malawi, Chad and Ethiopia [6], [7], [8]. Ethiopia has taken the OTP as most important and accessible program to treat malnutrition. It is operational at health centers and health posts and offers the lifesaving treatments with ready-to-use therapeutic foods (RUTF), usually Plumpy'Nut. Plumpy'Nut is energy, mineral and vitamin enriched paste-food designed to treat SAM (Table S1). A sachet has a serving size of 92 gm and gives energy of 2,100 kJ (500 kcal). In addition to Plumpy'Nut, severely malnourished children are provided with routine medications such as de-worming tabs, antibiotics, vitamin A, folic acid and measles vaccine. Only children who have appetite with Plumpy'Nut and those who don't have medical complications are eligible to the OTP [9]. This group of children represents over 90% of all SAM cases. With adequate coverage of OTP, hundreds of thousand child deaths can be alleviated each year thus contributing to the achievement of child mortality reduction in millennium development goals [3].

In Ethiopia, the program was piloted in specific areas in 2004 but now expanded to every health center and health post of the country [10]. Despite the existence of OTP and other nutrition programs in every corner of the country, the national survey and different studies have showed that malnutrition is indicated to be still high [5]. The Ethiopia Demographic and Health Survey reported that stunting, wasting and underweight are very high [4]. In addition, studies conducted in south Ethiopia and Jimma-south west Ethiopia- revealed that the recovery rate and defaulter rate were remote from the international acceptable standard ranges [11], [12]. This implies that investigating a study is important to obtain evidences regarding the program effectiveness. OTP program effectiveness is gauged by the global sphere standards.

Little is known about the outcomes level and factors determining the recovery rate of the children from SAM. Hence, we aimed to fill these gaps by assessing the outcomes (recovery, default, non-respondent and mortality rates) and estimating the length of intervention period to reach the minimum sphere standard recovery rate among the cohort of 6–59 months age SAM children relying on their individual OTP record cards in Tigray region, Northern Ethiopia. Moreover, we compared the different OTP outcome levels between the children with and without medical problems and analyzed the level of contribution of demographic characteristics, Plumpy'Nut, routine medications and clinical features to the recovery rate.

## Methods

### Study design and settings

A retrospective cohort study was conducted among children aged 6–59 months who had been treated for SAM under the OTP from April 2008 to January 2012. The research was carried out in Tigray region, northern Ethiopia. Tigray region is one of the nine regions of Ethiopia which is deemed the most drought-prone. The under-five population of the region is estimated to be 629,517 according to the 2007 Ethiopian census [13].

The Outpatient Therapeutic Feeding Program is delivered at health centers and health posts to treat severely malnourished children in their catchment area. The study zone owned 39 health centers and 107 health posts. A health center is primary health care unit staffed with midlevel health professionals such as nurses, midwives, public health professionals and laboratory technicians. It renders out-patient curative and preventive health services to about 25,000 people. Within its catchment area, there are five satellite health posts- the lowest tier in the Ethiopian health system hierarchy- each serving to 5,000 people. The five health posts are accountable to the health center. A health post is built in each sub-district and is employed with two female health extension workers (HEWs). The HEWs are mainly responsible for home-to-home prevention, diseases control and promotion of health care services [14]. In addition, they provide ante natal care, delivery, post natal care, family planning, immunization services, management of severe malnutrition, and growth monitoring and promotion [15].

### Outpatient Therapeutic Feeding Program (OTP)

The OTP offers services to severely malnourished children age 6–59 months. According to the protocol for management of SAM, Mid Upper Arm Circumference (MUAC) of less than 110 mms and/or weight-for-height ratio of less than 70% or presence of bilateral pitting edema are the eligibility admission criteria into the OTP. Regardless of these, children presented with medical problems won't be admitted to the OTP because health centers don't have inpatient services. Rather, they need to be referred to Therapeutic Feeding Units (TFU). The TFU is operational in hospitals which treats severely malnourished children with medical comorbidities as inpatient at least until their illnesses get stabilized. The medical problems indicated for referral are fever >37.5°C, bloody or persistent diarrhea, persistent vomiting, open skin lesions, loss of appetite with Plumpy'Nut and dehydration [16]–[18].

Once admitted to the OTP, children get a weekly Plumpy'Nut ration. They receive different amount of Plumpy'Nut sachets according to their body weight. They are also supplemented with the routine medications during the course of the treatment such as Vitamin A, Folic acid tabs, antibiotics, de-worming tabs and measles vaccine. Children admitted with marasmus cases get discharged from the OTP when they reach target weight and/or weight-for-height ratio >85%. Unlike the marasmus cases, the Kwashiorkor cases are discharged from the OTP after their edema gets disappeared regardless of their body weight status. These children are declared as ‘recovered’. Nonetheless, the children may have different outcomes such as ‘defaulter’, ‘non-respondent’, ‘medical transfer’ and ‘died’. ‘Defaulter’ is a patient that is absent for two consecutive weeks and confirmed that the patient is not dead by home visit. If the patient is confirmed as dead by home visit, s/he is labeled as ‘died’. A patient that has not reached either of the discharge criteria after staying under OTP intervention is determined to be ‘non-respondent’. A patient is determined as ‘Medial transfer’ when s/he develops any medical complications and referred to hospital for treatment under TFU.

### Study population and sampling technique

**Figure 1:**
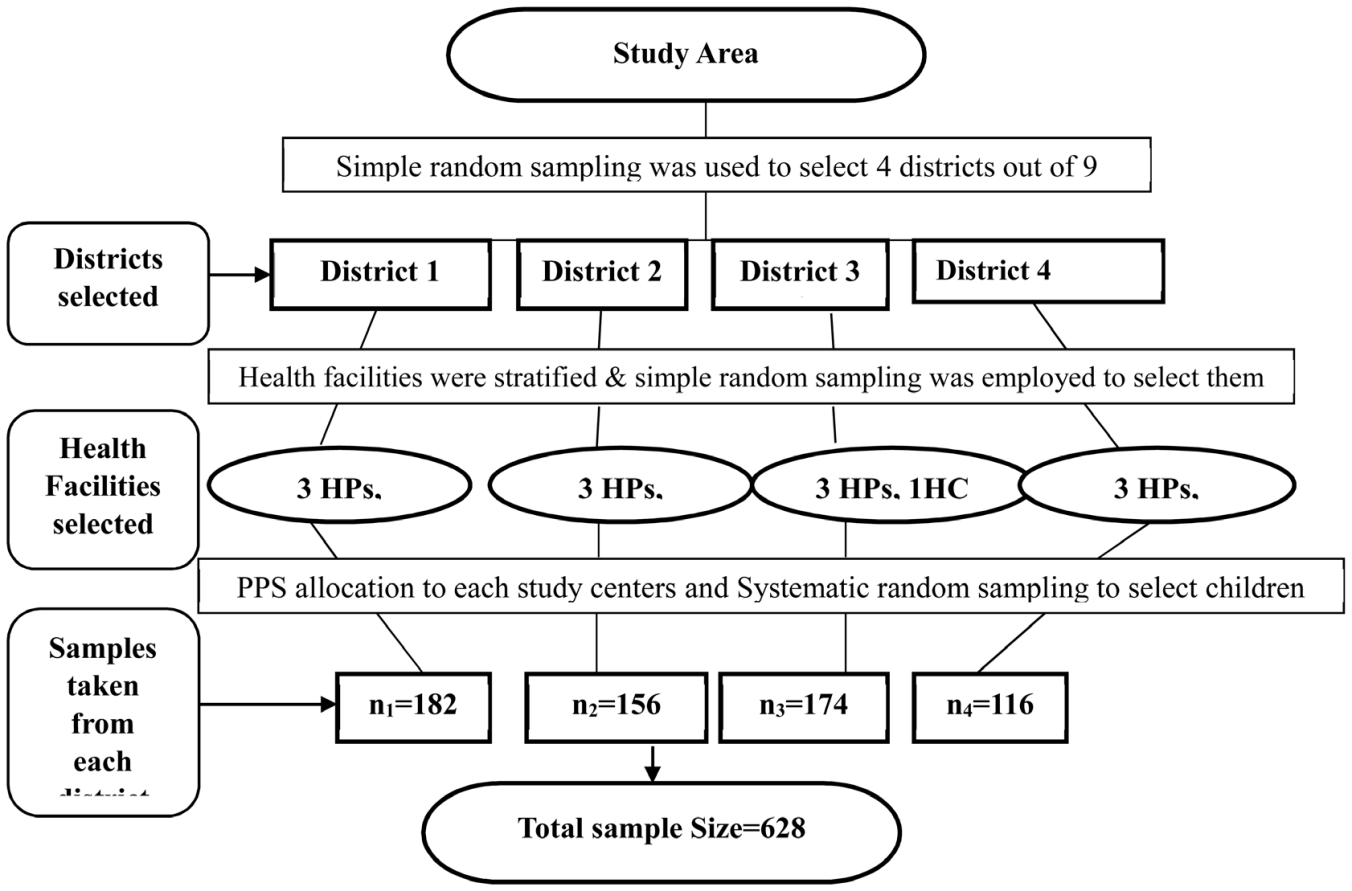
Sampling procedures. The phenomena regarding the child nutrition were assumed to be homogenous among the districts of the study zone. Thus, four districts out of nine were selected using simple random sampling. The health facilities rendering OTP were stratified into health centers and health posts. One health center and three satellite health posts were included from each district. Using the Probability Proportional to Size (PPS), the n1, n2, n3, and n4 samples were drawn. Finally, the OTP record card of each child was selected using systematic random sampling. *HP: health post; HC: health center*.

The sample size was calculated using STATA version 11(Stata Corporation, College Station, TX, USA) based on assumed population parameters. The significance level, power, Hazard Ratio (effect of taking amoxicillin on recovery rate as compared to not taking) were 5%, 80% and 1.25 respectively. Hence, a sample size of 628 individual OTP records of children managed for SAM was estimated for this study. In the study zone, there were nine districts. These districts were deemed more or less homogenous with characteristics related with child nutrition such as the health coverage of the areas, harvesting, and accessibility to information. As the result, four districts were selected at random using lottery method presuming that there was no information lost with the unselected districts. All OTP running institutions in the sampled districts were stratified into health centers and health posts. This was because the settings of health center and health post were different with regard to staffing and distance to the users which in turn might have effect on OTP outcomes differences. The under-five population managed in each sampled health institutions was assessed and it outnumbered in health centers than health posts. By the fact that health centers within a district have similar settings, we selected one health center from each district using simple random sampling. Out of the five satellite health posts under the catchment area of the health center, we included three health posts selected randomly using lottery method. In total, we prepared a sampling frame of children managed for SAM from four health centers and twelve satellite health posts in the four districts. Samples were allotted to each health institution using the probability proportional to sampling. Finally, the children were selected by systematic random sampling from each institution based on their unique identification number (Figure 1).

### Data Collection Procedure and Data Quality Control

The source of data for the study was individual OTP record cards. The cards consisted of information recorded at admission such as distance from home to the OTP center, sex, age of the child, anthropometry measurements, admission medical history, physical examination and routine medications. The cards also recorded the amount of Plumpy'Nut ration provided to the children in each week until they get discharged, follow-up anthropometry measurements and clinical features, routine medications and outcome status. All these data were collected using a uniform extraction format. Few information such as the level of maternal educational and consumption of the Plumpy'Nut at home are not indexed in the OTP record cards.

We recruited 10 nurses and 6 health extension supervisors for data collection. Five supervisors for the data collection were also recruited from intern medical students. To keep the quality of data, we trained the data collectors and supervisors for a day at each facility on how and what information they should be collecting from the OTP record cards. On daily basis, the filled-in information extraction formats were reviewed by the supervisors and investigators to scrutiny any problems. Whenever there appears incompleteness, errors, and ambiguities of recording, the information formats were crosschecked with the source card on spot. Those transferred-in and transferred-out children during the treatment were excluded from the study because we couldn't find full information.

### Statistical analysis

Data was entered into, cleaned and analyzed using STATA version 11. Exploratory data analysis was carried out to check the levels of missing values, presence of influential outliers, multi-collinearity, normality and proportionality of hazards over time. The missing values were less than 6% in almost all factors. However, the distance of home to the OTP site was missed in 35% of the cards; thus, it was excluded from the Cox regression model. Height was excluded from the multivariate analysis since it was highly collinear with age of the children. The presence of edema was very rare with an expected value of less than 5. It was, then, excluded from Cox regression model.

The proportion of the OTP outcomes were reported as ‘recovered’, ‘died’, ‘non-respondent’, ‘defaulter’ and ‘medical transfer’ using numbers and percentage. The program outcomes were also tabulated against the presence of medical complications. The average length of stay was also computed between the children with and without medical complications.

As to the international sphere standard, the length of stay under the OTP and rate of weight gain were computed only for the children recovered from SAM and those admitted with marasmus cases. Children who were admitted with Kwashiorkor were excluded form computing the rate of weight gain regardless of their recovery status. We computed the rate of weight gain with the formula:
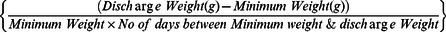
Children get discharged from the OTP at any time they reach the discharge criteria; all don't stay for the same intervention period under the OTP. As a result, Cox regression method was used to model the level of contribution of demographic characteristics, Plumpy'Nut, routine medications and clinical features to time-to-recovery from SAM. We coded the outcome into two-‘recovered’ and ‘not recovered’ and used Multivariate Cox-regression to determine the average hazard ratio by controlling the confounding effect of each factors. Noted that the survival rate in this study is contextualized for the recovery rate i.e. the outcome of interest is the time to recover from SAM. The recovery rates of the children from SAM were illustrated using Kaplan Meier (KM) survival curves for the grouped factors over the intervention period. To assess the significance of any difference observed in survival curves between the grouped factors, we employed the log-rank test. Any statistical test was considered significant at P<0.05. Finally, we checked the Cox regression model for its fitness to the data and its adequacy.

### Ethical statement

The study used the routine existing OTP data. Ethical approval was obtained from Mekelle University, College of Health Sciences with reference number CHS/1861/DN/16. The study was conducted in collaboration with the program lead-Tigray Regional Health Bureau, which gave us a support letter referred with 1508/334/04. Consents were also obtained from respective district health offices and health facilities. In the existing system of the nation, there is no way to communicate with the patients once they get discharged from the treatment. As such, informed consent from the parents/caregivers of the children was not obtainable. The study didn't give any supplementary interventions to the participants. As it was conducted based on the OTP records and was not sensitive, the consent relied on the ethical board's approval and written consent from respective district health offices and health facilities. Furthermore, the research ethical board was also aware of the issue before its approval that informed consent couldn't be obtained from the study participants. To append, any patient identifying information wasn't encoded to keep the confidentiality of the information.

## Results

### Characteristics of children

The study included 628 eligible children who had been managed for SAM under the OTP. Majority of the children, 618(98.40%), were admitted with marasmus and the rest with Kwashiorkor. Children beyond two years of age, 83(13.21%), were underrepresented in the OTP as compared to their younger age groups, 535(86.79%). The median age of the children at admission was 11 months (Inter quartile range: 11 to 17 months).

### Medical complications and OTP treatment outcomes

After the OTP intervention, children showed a 21.4% weight increase during discharge as compared with their admission weight (paired t-test = 1.31 Kgs, P<0.0001). The average distance from home to the OTP center in health center and back to home was 96.07 minutes on foot. The health posts are built more decentralized than health centers and the to-and-from distance was 64.63 minutes on walk.

Though the standard protocol for management of SAM does not allow to, the study explored that 279(44.3%) of the children with at least one medical problem were managed under the OTP. The most frequent medical problem was diarrhea, 212(33.75%) and least was fever 51(6.4%). The OTP providers made medical transfer/referral only to 5.41% of the children. Diarrhea, vomiting, cough and fever were reported unclassified for their types, severities and magnitudes; thus it was confronting to decide whether the children should have been referred to TFU or not. Nonetheless, loss of appetite with the Plumpy'Nut and failure to gain any weight for at least three consecutive weeks of intervention were reported in 81(10.10%) and 143(17.90%) of the children respectively. Both medical problems were clear indication for referral to TFU but the children were inappropriately managed under the OTP (Table 1).

**Table 1:** The medical problems identified during the OTP treatment, 2008–2012, Tigray, northern Ethiopia.

Medical Complications	N	▪ Proportion of medical problems	□ Proportion of Children with medical problems
Failure to gain any weight for ≥3 weeks	143	17.9%	22.77%
Diarrhea<$>\raster(70%)="rg1"<$>	212	26.5%	33.75%
Vomiting<$>\raster(70%)="rg1"<$>	193	24.1%	30.72%
Cough<$>\raster(70%)="rg1"<$>	121	15.1%	19.26%
Appetite Test Failure	81	10.1%	12.89%
Fever<$>\raster(70%)="rg1"<$>	51	6.4%	8.12%
Total	801	100%	

^<$>\raster(70%)="rg3"<$>^ *The medical problems were reported unclassified for their types, magnitude and severities.*

▪ *The proportion of each medical problems out of all (the denominator is the children the medical problems).*

□ *The proportion of children who had medical problems (the denominator is the total children in the study).*

The study showed that 388(61.78%) of the children who had been managed for SAM were recovered after treated under the OTP intervention. The OTP treatment outcomes were different between the children with and without medical problems. Children without medical comorbidity were with better recovery rate than those with medical problems, 293(83.95%) versus 95(34.05%). The children recovered from SAM had gained an average weight of 5.24 gm/kg/day (95% CI = 4.98, 5.63). However, the rate of weight gain among the children without any medical complications was 6.30 gm/kg/day which was higher than those with at least one medical complication, 4.16 gm/kg/day (Table 2).

**Table 2:** The comparison of the study results with international Standards, 2008–2012, Tigray, northern Ethiopia.

	Results			Sphere Standards	
Indicators	Children Without Medical Problems	Children With Medical Problems	Overall	Acceptable	Alarming
Proportion of children who recovered from severe acute malnutrition after treated in OTP ***(Recovery rate)***	83.95%	34.05%	61.78%	>75%	<50%
Proportion of severe acute malnourished children who died while under the OTP intervention ***(death rate)***	0%	6.81%	3.02%	<10%	>15%
Proportion of severe acute malnourished children who defaulted from the OTP ***(defaulter rate)***	2.57%	27.98%	13.85%	<15%	>25%
The average rate of weight gain (*gm kg^−1^ child^−1^ day^−1^*) ***(rate of weight gain)***	6.30 (6.09, 6.48)	4.16 (3.89, 4.42)	5.23 (4.98, 5.63)	> = 8	<8
Number of weeks that the children stay under the OTP ***(average length of stay)***	6.24 weeks	6.25 weeks	6.24 *weeks*	<4 *weeks* <8weeks???	>6 *weeks*

^<$>\raster(70%)="rg2"<$>^ *Unlike the sphere (international) standard which set to be 4–6 weeks, the Ethiopian protocol for management of SAM indicated for children to say in the OTP for a maximum of eight week.*

The defaulter rate was 87(13.85%) and the average defaulting time was 3.34weeks. The children with medical comorbidities accounted for 90% of the defaulting rate. Nineteen (3.02%) of the children died while they were being treated under the OTP intervention and all of them had at least one medical problem (Table 2). The mean length of stay under the intervention was 6.48weeks (95% CI = 6.25, 6.72). Though the Ethiopian protocol for management of SAM limit the maximum length of stay under the treatment to 8 weeks, the OTP service providers in this study retained 87(13.90%) of the children for more than 8 weeks (8–12weeks) under the OTP. No matter for how long they stayed under the OTP, the children who didn't reach any of the discharge criteria (non-respondents) were 56 (8.91%).

More or less, the recovery and non-respondent rates were similar in the health centers and health posts except the mortality rate showed a slight increase in health center (7.80%) as compare to health post (6.05%).

### Routine medicines administration

Under the OTP, according to the standard, children need to be administered routine medicines together with the Plumpy'Nut. In this study, all children had taken Plumpy'Nut. However, 139 (22.1%) of the eligible children did not receive at least one of the routine medications. The rest of the children, 489(77.9%), had received the routine medications partially. The most administered medications were amoxicillin (72.13%) and Vitamin A (59.17%) while the least was Folic acid which was administered to only (5.89%) (Table 3).

**Table 3:** Routine medications intake among eligible children managed under OTP, 2008–2012, Tigray, northern Ethiopia

Routine Medicines *(n = eligible children)*	N	▪ Proportion of medication intakes	□ Proportion of children who took drugs
Amoxicillin *(n = 628)*	453	36.0%	72.13%
Vitamin A *(n = 628)*	371	29.4%	59.17%
De-worming *(n = 354)*	193	15.3%	54.51%
Measles vaccine *(n = 495)*	206	16.3%	41.61%
Folic Acid *(n = 628)*	37	2.9%	5.89%
Total	1260	100%	

▪ *The proportion of each medication administered out of all medications (the denominator is the total medication administered)*.

□ *The proportion of children who took the each medication (the denominator is the total eligible children in the study).*

### Summary of recovery rates and testing for statistical differences

**Figure 2:**
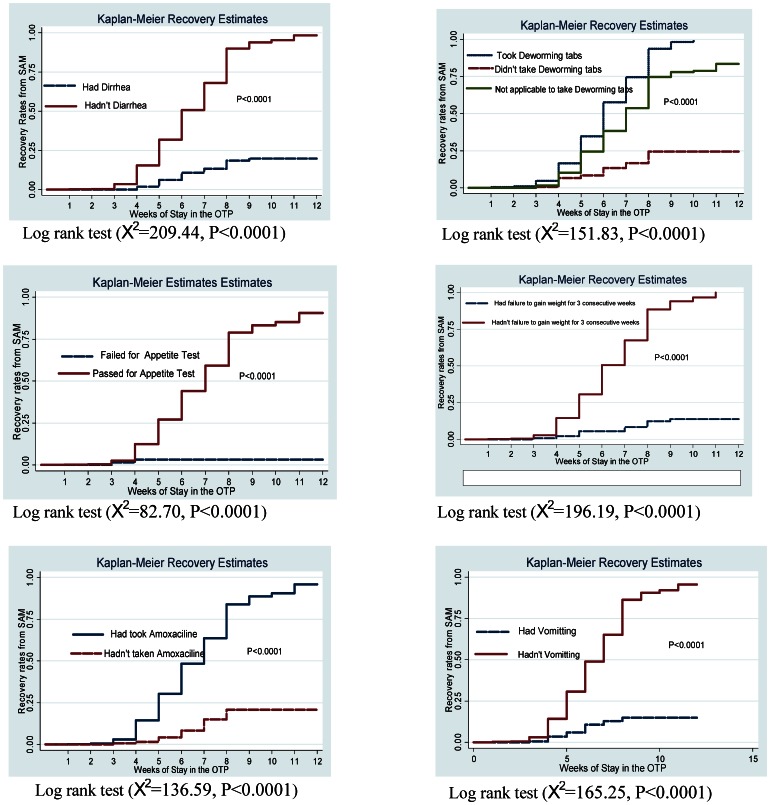
Kaplan Meier survival curves and Log-rank test for recovery rates over grouped factors. The KM survival curves for each grouped factor were identified by color and pattern differences. They showed the recovery rates over the OTP intervention. The KM curves enable to compare the recovery rates between those with and without diarrhea, vomiting, loss of appetite with Plumpy'Nut, failure to gain weight and over children who took de-worming and amoxicillin drugs as compared to those who didn't take the drugs. The log-rank tests the significance of the observed differences in recovery rates on the KM survival curves between the grouped factors. *X^2^: Chi-squared test*.

The rate of recovery from SAM among the children under the OTP was 99/1000 children/week. The median recovery time was 7 weeks i.e. 50% percent of the children recovered from SAM at less than 7 weeks. Likewise, to reach the minimum sphere standard recovery rate, set at 75%, the children were demanded 9 weeks to stay under the treatment which was 5 weeks beyond the standard. However, less than 8 weeks was sufficient for the children without medical comorbidities and to those who received routine medications to recover from SAM. Not a small number of children were managed inappropriately under the OTP and this had negative effect on the recovery rate. Children who didn't receive routine medications and those with medical problems didn't attain recovery rate of 25% even they stayed under the intervention for 8 weeks and more. To show the recovery rates during the courses of intervention period, we depicted the Kaplan Meier survival curves over different grouped factors. Generally in all the cases, children managed inappropriately had lower recovery rate per week. That is, children with diarrhea, vomiting, loss of appetite with, failure to gain weight for three consecutive weeks and those who didn't receive routine drugs had consistently lower recovery rate per week over the intervention period (Figure 2).

To test the significance of the observed difference in survival (recovery rate) curves between each grouped factors, the log-rank test was employed. There was significantly different recovery rates among children with and without diarrhea (Log-rank test, 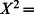
209.44, P<0.0001), vomiting (Log rank test, 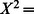
165.25, P<0.0001), loss of appetite test with Plumpy'Nut (Log-rank test, 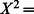
82.70, P<0.0001), failure to gain weight gain for at least 3 consecutive weeks (Log-rank test, 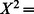
214.42, P<0.0001). In summary, the recovery rate from SAM per week was lower and significantly different among the children with diarrhea, vomiting, loss of appetite with Plumpy'Nut, failure to gain weight than the children without these medical problems. Likewise, eligible children who received amoxicillin and de-worming drugs exhibited higher and significantly different recovery rates during the intervention period as compared to those who didn't receive the drugs, Log-rank test, 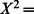
136.59, P<0.0001 versus Log-rank test, 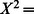
151.83, P<0.0001 (Figure 2). In light of these, complying with the appropriate management of OTP resulted to significantly faster recovery rate.

### Multivariate Cox regression

Children get discharged from the OTP the sooner time they achieve the discharge criteria; all don't stay for 8 weeks under the OTP. Thus, modeling time-to-recovery is more advantageous than modeling the recovery status without time determinant. To reveal the effect levels of independent variables to recovery rate (survival) from SAM, Multivariate Cox-regression was computed over the age of children, sex, Plumpy'Nut, admission status, medical problems, routine medications, type of health facility by controlling their confounding effect. With these factors, the final model was very highly significant to predict the survival (recovery) rate of children from SAM after the OTP intervention, 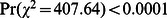.

Plumpy'Nut had a positive effect to the recovery rate (Hazard ratio-HR = 1.04, 95% CI = 1.03, 1.05). As a child consumed one more sachet of Plumpy'Nut, the recovery rate from SAM increased by 4%. Presence of diarrhea, vomiting, failure to gain weight for at least 3 consecutive weeks, appetite loss with Plumpy'Nut, average weight gain, amoxicillin and de-worming drug intakes were significant to predict the recovery rate from SAM. Yet, whether a child was admitted to the OTP as new or defaulter, sex, age, the site of OTP service didn't have significant effect on the recovery rate. Thus, they were excluded from the final model. Controlling for other factors, the patients with no diarrhea at a point of time during intervention had 2.2 times higher probability of getting recovered from SAM by next visit as compared to the patients with diarrhea (HR = 2.20, 95% CI = 1.31, 3.41, P<0.01). Likewise, the children admitted without vomiting at a point of time during the treatment had 2.1 times higher probability of getting recovered from SAM by next visit as compared to those with vomiting (HR = 2.13, 95% CI = 1.20, 3.78, P<0.01). The hazard ratios of loss of appetite with Plumpy'Nut and failure to gain weight were 4.49(95% CI = 1.02, 19.62, P<0.05) and 3.88(95% CI = 2.14, 6.39, P<0.0001) respectively. Children who didn't have these problems were with higher probability of getting recovered from SAM as compared to those with the problems. Furthermore, the probability of getting recovered from SAM at a time among the children who took amoxicillin and de-worming drugs had 95% (HR = 1.95, 95% CI = 1.17, 3.23, P<0.05) and 74% (HR = 1.74, 95% CI = 1.07, 2.83, P<0.05) more as compared to those who didn't take them respectively (Table 4).

**Table 4:** Multivariate Cox-regression for prediction of recovery rate from SAM 2008–2012, Tigray, northern Ethiopia.

Factors	Category	Number (%)	Adjusted Hazard Ratio	95% Conf. Interval
Average Plumpy'Nut intake	*N/A*	16(15.63, 16.34)	1.04***	1.03, 1.05
Diarrhea at admission or during	*Yes*	213(33.92)	1.00	
follow up	*No*	415(68.08)	2.20**	1.31, 3.41
Vomiting at admission or during	*Yes*	193(30.73)	1.00	
follow up	*No*	435(69.27)	2.13**	1.20, 3.78
Failure to gain weight for 3 con-	*Yes*	143(22.77)	1.00	
-secutive weeks	*No*	485(77.23)	3.88***	2.14, 6.39
Appetite Test with Plumpy'Nut	*Failed*	81(12.90)	1.00	
	*Passed*	547(87.10)	4.49*	1.03,19.62
Amoxicillin intake	*Yes*	453(72.13)	1.95*	1.18, 3.10
	*No*	175(27.87)	1.00	
De-worming Tabs intake	*Yes*	193(30.73)	1.74*	1.07, 2.83
	*No*	161(25.64)	1.00	
	*N/A+*	274(43.63)	1.88*	1.05, 3.37

* *Significant at P<0.05,*

** *significant at P<0.01,*

*** *significant at P<0.001.*

*N/A: Not applicable and N/A^+^ not applicable i.e. children less than one year ages are not eligible to take de-worming tabs).*

*HR = Hazard ratio.*

*All the predictors in the table were adjusted for one another to control for confounding effect.*

We recognized from this study that the health workers didn't comply with the standard for OTP management. As such, the Multivariate Cox-regression pointed out that having medical complications at admission or during intervention and not taking the routine drugs had negative effect to the recovery rate.

## Discussion

The study revealed that the defaulter and mortality rates were within the acceptable international sphere standards. But, the recovery rate and rate of weight gain were below the standards. Children with medical comorbidities were managed inappropriately under the OTP intervention and large proportion of children was administered routine drugs partially.

The overall mortality rate (3.02%) is well within the minimum sphere standard (<10%); but it's slightly higher than similar studies conducted in Jimma and Arbegona, Ethiopia [12], [19], [20]. The mortality rate was zero among the children without medical problems as compared to those with medical problems. OTP providers didn't comply with SAM management protocol. As evidence, OTP routine medications were provided to the children partially. Children with medical problems, who are indicated to inpatient treatment in hospitals, were managed under the OTP (Table 1, Table 3). Hypothetically the magnitude of the mortality is expected to be higher as many unknown cases, which might have died at home, have remained untraced. In addition, the indexed child might not be forced to eat the food and there might be sharing of the Plumpy'Nut among other siblings at home. These improper states of management may contribute to the increased mortality rates of the children.

The defaulter rate in this study is within the acceptable range of the international standard. This is contained in the domain of global sphere standards (Table 2). The children with medical comorbidities were defaulting at much higher rate. The overall defaulter rate in this study is higher as compared to similar study conducted on RUTF based therapeutic feeding program in Maradi-Niger, Darfur-Sudan, Bedawacho-Ethiopia and Arbegoba-Ethiopia [20]–[23]. This might be because the therapeutic feeding program in Maradi and Darfur was introduced in response to emergency situation (hunger gap) and might get especial focus. For instance, children admitted to the OTP in Darfur, Sudan were visited daily by the community Nutrition workers who checked the child for appetite, diarrheal history, thirst and dehydration. They confirmed the presence of a caregiver and watched the child consume Plumpy'Nut in addition to the full medical examination [22]. Moreover, the caregivers in those areas could adhere to the treatment since other foods are scarce at home during famine [21]. However in Tigray-Ethiopia, the program is implemented as one of the routine medical services regardless of the non-existence of drought and it might not get same focuses as in hunger situations. This result signifies that applying strict follow ups on the children under treatment could result to better outcomes.

The average length of stay under the OTP intervention was 6.24weeks (43.68 days). This is by far outside of the acceptable minimum international standard (<28 days) [24]; yet it is well within the standard of the Ethiopian protocol for management of SAM which allows children to stay under treatment to utmost 8weeks (64 days) [23] (Table 2). This length of stay is comparable to other similar studies of OTP outcomes evaluation conducted in Bedawacho-Ethiopia (42 days) and Maradi-Niger (42.6 days) [21], [23]. However, there is delay not far off a week in the OTP treatment in this study as compared to similar study conducted on evaluation of OTP in Jimma-Ethiopia (37.5 days) [11]. The estimated length of stay could be inflated because 13.90% were tolerated to stay for 8–12 under the intervention though they were non-respondent. That is, these children should have been referred to hospitals for inpatient treatment under TFU at their 8^th^ week of stay under the OTP when they failed to reach any of the discharge criteria [17], [25]. The partial administration of routine medications and managing children with medical compilations might also contribute to the delay of recovery from SAM.

In this study, recovery rate was estimated for all children no matter for how long they stayed under the OTP. The recovery rate was (61.78%) and it is lower than the international standard which bars the lower threshold at 75% (Table 4). As well, it was lower than findings from studies in Maradi-Niger and Darfur-Sudan [21], [22]. The average rate of weight gain was substantially less than the predicted rate based on the international standard and the findings in Darfur-Sudan and Maradi-Niger [21], [22]. More importantly, the recovery rate and rate of weight gain were much higher among the children without medical problems. Violating the standard protocol for SAM management affected the recovery rate and weight gain negatively. These discrepancies could also be resulted from sharing of the Plumpy'Nut with other members of a household or the caregivers might not force the index child to consume the right amount of the food.

The length of stay under the OTP has social, psychological and economic implications. Hence, globally it is set it to be less than 4–6 weeks considering that at least 75% of the children at this point could get recovered from SAM. The Ethiopian Ministry of Health also customized this and fixed it to be utmost 8 weeks. The length of stay needed to reach the minimum international standard recovery rate was higher in this study. Inappropriate management of the OTP accounted for these delayed rates. We endeavored to demonstrate the effect of inappropriate management to recovery rate using the Kaplan Meier survival curves and Log-rank test. It was pointed out that children with medical comorbidities and those who didn't take routine medications had significantly lower recovery rate per week as compared to the children without medical problems and those took routine drugs. These findings imply us that only by complying with the standard protocol for management of SAM, better program outcomes could be assured (Figure 2).

The Multivariate Cox-regression showed that taking amoxicillin, de-worming drugs and Plumpy'Nut were positive predictors to recovery rate from SAM. But, having diarrhea, vomiting, loss of appetite with Plumpy'Nut and lose to gain weight for at least three consecutive weeks were negative predictors to recovery rate from SAM (Table 4). The following explanations could substantiate for the aforementioned factors. Despite the absence of clinical signs, severely malnourished children are nearly all infected, particularly if they have poor appetite. All children might not show clinical signs and symptoms of systematic infections as the result of their low body immunity. However, small bowel bacterial overgrowth occurs in all these children (including those with moderate, and some with good appetites) [26]. These enteric bacteria might be frequently the source of systemic infection by translocation across the bowel wall. They also cause mal-absorption of nutrients, failure to eliminate substances excreted in the bile, fatty liver, intestinal damage and can cause chronic diarrhea [24]. The severely malnourished children are also the most harbors of parasites which can directly consume nutrients and/or deter the absorption. Hence, these infections should be treated blindly. Yet, this study revealed that all eligible children were not administered the antibiotics and de-worming [10], [25].

The study is substantiated with better design and analysis methods. It has assessed different factors that might affect the overall program effectiveness. The findings could be strong and reliable to apply for the general children population under treatment. However, this study might have a limitation in that it did not assess the information on treatment compliance at home-level such as existence of sharing of Plumpy'Nut among siblings, proper provision of the treatment to the indexed child at home and effect of the maternal educational level. This information is not indexed in the standard OTP record cards. Indeed, the effect might not be vigorous to delude the findings; because in practice, there is an indirect way to check the consumption of Plumpy'Nut by the child. There might be inaccuracy in measurement of MUAC during admission which might lead to misclassification of children to be included in or exclude from OTP.

In conclusion, the overall recovery rate and rate of weight gain were lower than the sphere standards; but, the defaulter and mortality rates were within the acceptable range of sphere standards. Thus, the OTP program was partially effective. Management of children with comorbidities under the OTP and the partial administration of routine medications were major threats for the program effectiveness. The stakeholders should focus on creating the capacity of the OTP providers on proper management of severe acute malnutrition to achieve fully effective program. To complement the aforementioned potential limitations, further studies need to reveal the treatment situations at home-level such as sharing of Plumpy'Nut with siblings and maternal education.

## Supporting Information

Table S1 Nutritional composition of a 100 g of Plumpy'Nut.
(DOC)
