# Supplementary material for: Outpatient Therapeutic Feeding Program Outcomes and Determinants in Treatment of Severe Acute Malnutrition in Tigray, Northern Ethiopia: A Retrospective Cohort Study
Source: PLoS One. 2013 Jun 6;8(6):e65840. doi: 10.1371/journal.pone.0065840 (PMC3675046; doi:10.1371/journal.pone.0065840)
Supplement: Table S1 — Nutritional composition of a 100 g of Plumpy'Nut. (DOC) [file pone.0065840.s001.doc]

Table S1: The nutritional composition of a 100g of Plumpy'Nut

| **Protein, Energy, Vitamin A and Minerals** | **Amount** |
| --- | --- |
| Energy | 2.29 MJ |
| Protein | 13.3 g |
| Lipid | 35.3 g |
| Calcium | 320 mg |
| Phosphorus | 394 mg |
| Potassium | 1111 mg |
| Magnesium | 92 mg |
| Copper | 1.78 mg |
| Iron | 11.53 |
| Iodine | 110 $\mu$g |
| Sodium | 189 mg |
| Zinc | 14 mg |
| Selenium | 30 $\mu$g |
| Vit A | 910 $\mu$g |
| Vit D | 16$\mu$g |
| Vit E | 20 mg |
| Vit C | 53 mg |
| Vit B1 | 0.6 mg |
| Vit B2 | 1.8 mg |
| Vit B6 | 0.6 mg |
| Vit B12 | 0.53 $\mu$g |
| Vit K | 21 $\mu$g |
| Biotin | 65 $\mu$g |
| Folic Acid | 210 $\mu$g |
| Pantothenic acid | 3.1 mg |
| Niacin | 5.3 mg |
